# Risk factors analysis for significant liver fibrosis of prenatally diagnosed choledochal cysts: a retrospective case-control study

**DOI:** 10.3389/fped.2025.1595238

**Published:** 2025-05-13

**Authors:** XiaoJin Zhuang, YuanBin He, DianMing Wu, YiFan Fang, Yu Lin, YanBing Huang

**Affiliations:** ^1^ Department of Pathology, Fujian Children's Hospital (Fujian Branch of Shanghai Children's Medical Center), College of Clinical Medicine for Obstetrics & Gynecology and Pediatrics, Fujian Medical University, Fuzhou, China; ^2^ Department of General Surgery, Fujian Children's Hospital (Fujian Branch of Shanghai Children's Medical Center), College of Clinical Medicine for Obstetrics & Gynecology and Pediatrics, Fujian Medical University, Fuzhou, China

**Keywords:** risk factors, prenatal diagnosis, choledochal cyst, liver fibrosis, gamma-glutamyl transferase

## Abstract

**Background:**

The severity of liver fibrosis and optimal surgical timing in infants with prenatally diagnosed choledochal cysts (CDCs) remain contentious. This study aims to identify risk factors for significant liver fibrosis in prenatally diagnosed CDCs and guide optimal surgical timing.

**Methods:**

This retrospective case-control study reviewed infants with prenatally diagnosed CDCs between January 2016 and January 2024. Liver fibrosis was staged (S0–S4) using the Batts-Ludwig system. Infants were categorized into mild/no fibrosis (<S2) and significant fibrosis (≥S2) groups based on histopathology. The aspartate aminotransferase to platelet ratio index (APRI) and Fibrosis-4 index (FIB-4) were validated, and multivariate logistic regression analyses were performed to identify independent risk factors. The receiver operating characteristic (ROC) curve was used to assess diagnostic performance.

**Results:**

A total of 50 infants (20 male, 30 female) were enrolled, with a median gestational age at diagnosis of 28 weeks (range: 19–39 weeks) and a median surgical age of 54 days (range: 7–360 days). Liver fibrosis was present in 40 cases (80%), with 34 cases (68%) classified as <S2 and 16 cases (32%) as ≥S2. Univariate analysis showed that infants with significant liver fibrosis had a higher proportion of clinical symptoms and Type IV CDCs, as well as elevated AST, TBil, DBil, GGT, WBC, and cysts width before surgery (*p* < 0.05). Multivariate logistic regression analysis identified Type IV CDCs (OR = 11.39, 95% CI: 1.04–124.65) and GGT (OR = 1.003, 95% CI 1.00–1.01) as independent influencing factors (*p* < 0.05). For diagnosing significant fibrosis (≥S2), GGT demonstrated an area under the ROC curve (AUROC) of 0.86, with an optimal cutoff of 327 U/L (sensitivity: 75%, specificity: 88%). APRI showed an AUROC of 0.76 (95% CI 0.61–0.92, *p* < 0.01) with an optimal cutoff of 0.23 (sensitivity: 75%, specificity: 80%).

**Conclusion:**

Liver fibrosis is common in infants with prenatally diagnosed CDCs, primarily presenting as mild. Type IV CDCs and GGT > 327 U/L are significant risk factors, highlighting the need for close monitoring and timely surgical intervention.

## Introduction

Choledochal cysts (CDCs), also referred to as congenital bile duct dilatation, are rare congenital anomalies affecting the intrahepatic and extrahepatic bile ducts in pediatric populations, with an incidence of approximately 1 in 13,000 among Asian individuals (1). With advancements in prenatal diagnosis and care, up to 70% of CDC cases are identified during the fetal stage (1). Infants diagnosed prenatally with CDCs represent a distinct cohort, as they are at risk for early-onset liver fibrosis, necessitating prompt evaluation and surgical intervention. Previous studies had indicated that the prevalence of liver fibrosis in these infants ranges from 33% to 100%, with advanced fibrosis observed in 20% to 44% of cases (2–4).

Liver biopsy is currently the gold standard for evaluating liver fibrosis. However, it is invasive and associated with risks such as hemorrhage and sampling variability. Growing interest has emerged in non-invasive approaches for predicting liver fibrosis. The aspartate aminotransferase-to-platelet ratio index (APRI) and fibrosis-4 (FIB-4) index are widely used for non-invasive liver fibrosis assessment in both adults and children with viral hepatitis and biliary atresia (5–7). However, their validation in infants with prenatally diagnosed CDCs remains limited.

The optimal surgical timing and prognostic implications of liver fibrosis progression in infants with prenatally diagnosed CDCs remain controversial. This study aims to identify risk factors associated with significant liver fibrosis in pediatric cases and to evaluate the use of APRI and FIB-4 in assessing fibrosis severity.

## Materials and methods

### Patients' involvement

This retrospective case-control study was conducted at Fujian Children's Hospital from January 2016 to January 2024, involving 50 pediatric cases with prenatally diagnosed CDCs. The inclusion criteria were as follows: (1) prenatal ultrasonographic identification of choledochal cysts with postnatal confirmation via ultrasound or magnetic resonance cholangiopancreatography (MRCP), or intraoperative cholangiography; (2) complete cyst excision with Roux-en-Y hepaticojejunostomy; (3) acquisition of intraoperative liver biopsy specimens. Exclusion criteria encompassed: (1) inadequate availability of clinical records or absence of preoperative biochemical parameters; and (2) suboptimal histopathological specimen quality (defined as liver biopsy fragments <1.5 cm in length or compromised tissue architecture on hematoxylin-eosin staining).

### Data collection

Clinical data were retrospectively extracted from the institutional electronic medical record system, encompassing three principal categories: (1) Demographic and clinical characteristics (gender, gestational age at prenatal diagnosis, surgical age/weight, presenting symptoms); (2) Preoperative imaging parameters (Todani classification, cyst diameter); (3) Preoperative biochemical profiles (aspartate aminotransferase [AST], alanine aminotransferase [ALT], total bilirubin [TBIL], direct bilirubin [DBIL], gamma-glutamyl transferase [GGT], albumin [ALB], white blood cell count [WBC], platelet count [PLT]). All laboratory indices represented the most recent preoperative measurements preceding liver biopsy.

Fibrosis assessment was performed using validated formulae:

$$\begin{array}{c} \mathrm{APRI}:\;[\mathrm{AST}\;(U/L)/\mathrm{AST}(\mathrm{ULN})\;*\;100]/\mathrm{PLT}\;(10^{9}/L)\\ \mathrm{FIB}\text{-}4:\;\mathrm{Age}\;(\mathrm{years})\;*\;\mathrm{AST}\;(U/L)/[\mathrm{PLT}\;(10^{9}/L)\;*\;{\left(\mathrm{ALT}\;(U/L)\right)}^{1/2}] \end{array}$$

where ULN denotes the upper limit of normal reference values for AST.

### Liver pathology assessment

Liver histopathological evaluation was systematically performed on CDC patients using three standardized staining modalities: hematoxylin and eosin (H&E), Masson's trichrome, and reticulin staining. Fibrosis staging adhered to the Batts-Ludwig classification system (8): Stage 0: No fibrosis; Stage 1: Portal fibrosis without septa; Stage 2: Occasional bridging fibrosis; Stage 3: Marked bridging fibrosis; Stage 4: Established cirrhosis. Two independent pediatric pathologists from Fujian Provincial Children's Hospital conducted blinded histological evaluations, strictly segregated from clinical data analysis to ensure objective assessment. Stratification criteria: Non/mild fibrosis cohort (Batts-Ludwig stages 0–1); Significant fibrosis cohort (Batts-Ludwig stages 2–4).

### Statistical analysis

Statistical analyses were conducted using IBM SPSS Statistics (v26.0). Continuous variables with normal distribution were expressed as mean ± standard deviation (SD) and analyzed by independent Student's *t*-test; non-normally distributed data were presented as median (interquartile range, IQR) and compared using Mann–Whitney *U* test. Categorical variables were presented as number (%) and assessed using the appropriate chi-square test or Fisher's exact test. Multivariate logistic regression analysis was conducted to identify independent predictors. The diagnostic performance of continuous variables was evaluated by receiver operating characteristic (ROC) curve analysis, with optimal cutoff values determined by maximizing Youden's index. The area under the ROC curve (AUROC) quantified discrimination accuracy. Statistical significance was defined as two-tailed *p* < 0.05.

## Results

### Clinical characteristics of patients with prenatally diagnosed CDCs

**Figure 1:**
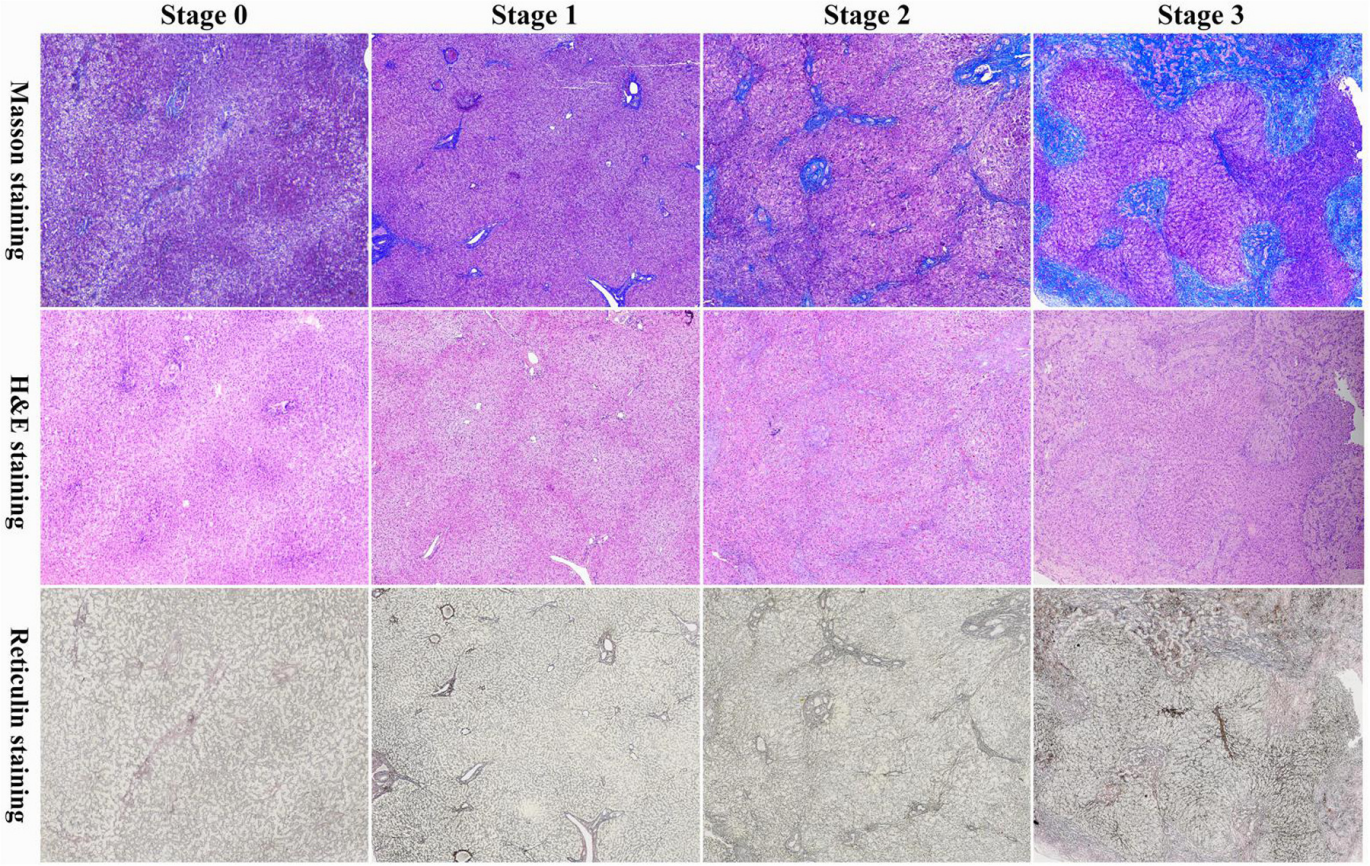
Masson's trichrome (masson), hematoxylin and eosin (H&E), and reticulin staining of liver fibrosis in infants with a prenatal diagnosis of CDCs. Batts-Ludwig Stage 0: no fibrosis; Batts-Ludwig Stage 1: expansion of the portal fibrosis area; Batts-Ludwig Stage 2: occasional formation of bridging structures and fibrous septa; Batts-Ludwig Stage 3: extensive formation of bridging structures and fibrous septa.

This cohort comprised 50 infants with prenatally diagnosed CDCs, with a male-to-female ratio of 2:3. Prenatal diagnosis was established at a median gestational age of 28 weeks (range: 19–39 weeks), and surgical intervention was performed at a median age of 54 days (range: 7–360 days). A total of 39 patients (78%) were asymptomatic, while 11 (22%) exhibited clinical manifestations, primarily jaundice in 5 cases, acholic stools in 3 cases, and emesis in 3 cases. Cyst classification revealed Type I CDCs in 39 cases (78%) and Type IV CDCs in 11 cases (22%), with a median cyst diameter of 3.3 cm (range: 1.2–9.3 cm) (Table 1). Liver fibrosis was present in 40 cases (80%), stratified as follows: S0: *n* = 10 (20%), S1: *n* = 24 (48%), S2: *n* = 11 (22%), S3: *n* = 5 (10%), with no cases of S4 (Figure 1).

**Table 1:** Clinical data of prenatally diagnosed CDCs, and comparison between mild/no fibrosis (<S2) and significant fibrosis (≥S2) group.

Factors	Total (*n* = 50)	<S2 (*n* = 34)	≥S2 (*n* = 16)	*p*
General data				
Gender, *n* (%)				
Male	20	11	9	0.111
Female	30	23	7	
Age at operation (day)	54 (7, 365)	54 (45, 113)	68 (30, 134)	0.860
Weight at operation (kg)	5.2 (2.8, 11.0)	5.2 (4.2, 5.9)	5.2 (3.6, 6.0)	0.795
Prenatal diagnosis (week)	28 (19, 39)	28 (24, 34)	34 (27, 34)	0.066
Symptomatic, *n* (%)				
Yes	11	3	8	0.001*
No	39	31	8	
MRCP				
Todani classification, *n* (%)				
I	39	31	8	0.002*
IV	11	3	8	
Cyst width (cm)	3.3 (1.2, 9.3)	2.9 (2, 4.0)	4.6 (3.3, 6.9)	0.007*
Laboratory parametersa				
ALT (U/L)	28 (7, 340)	28 (19, 40)	42 (19, 101)	0.183
AST (U/L)	39 (20, 44)	38 (34, 52)	78 (39, 140)	0.002*
TBIL (μmol/L)	38 (3, 359)	18 (9, 69)	78 (49, 198)	0.001*
DBIL (μmol/L)	7 (1, 183)	5 (2, 11)	39.5 (18, 55)	0.001*
GGT (U/L)	157 (7, 2,837)	70 (31, 239)	536 (283, 896)	0.001*
ALB (g/L)	40 (29, 50)	40 (38, 41)	40 (33, 430)	0.946
WBC(10^9^/L)	9.8 (5.6, 33.4)	9.7 (8.5, 11.5)	12.2 (9.3, 17.5)	0.019
PLT(10^9^/L)	402 (110, 710)	393 (310, 533)	405 (247, 490)	0.618
Non-invasive models				
APRI	0.19 (0.07, 2.48)	0.17 (0.11, 0.20)	0.37 (0.17, 0.78)	0.003*
FBI-4	0.003 (0.0.12)	0.003 (0.002, 0.006)	0.008 (0.002, 0.014)	0.140

Nonnormally distributed data was described as M (P25, P75). MRCP, magnetic resonance cholangiopancreatography; ALT, alanine aminotransferase; AST, aspartate aminotransferase; TBIL, total bilirubin; DBIL, direct bilirubin; GGT, gamma-glutamyl transferase; ALB, albumin; WBC, white blood cell count; PLT, platelet count.

* *p* value < 0.05.

At the final follow-up (median duration: 55 months; range: 6–120 months), all patients demonstrated normal growth parameters. Serum bilirubin levels and hepatic function markers normalized in all cases, and ultrasonographic assessment confirmed the resolution of preexisting intrahepatic bile duct dilation, with no residual ductal abnormalities.

### Univariate analysis of risk factors for advanced liver fibrosis in prenatally diagnosed CDCs

Univariate analysis identified significant differences in clinical presentation and pathophysiological parameters between groups. Patients with significant liver fibrosis had a higher prevalence of clinical symptoms (*p* < 0.05) and Type IV CDCs (*p* < 0.05) compared to those with mild fibrosis. Additionally, the significant fibrosis group exhibited elevated AST, TBil, DBil, GGT, and WBC levels, as well as larger cysts width (all *p* < 0.05) (Table 1).

### Multivariate analysis of advanced liver fibrosis of prenatally diagnosed CDCs

**Figure 2:**
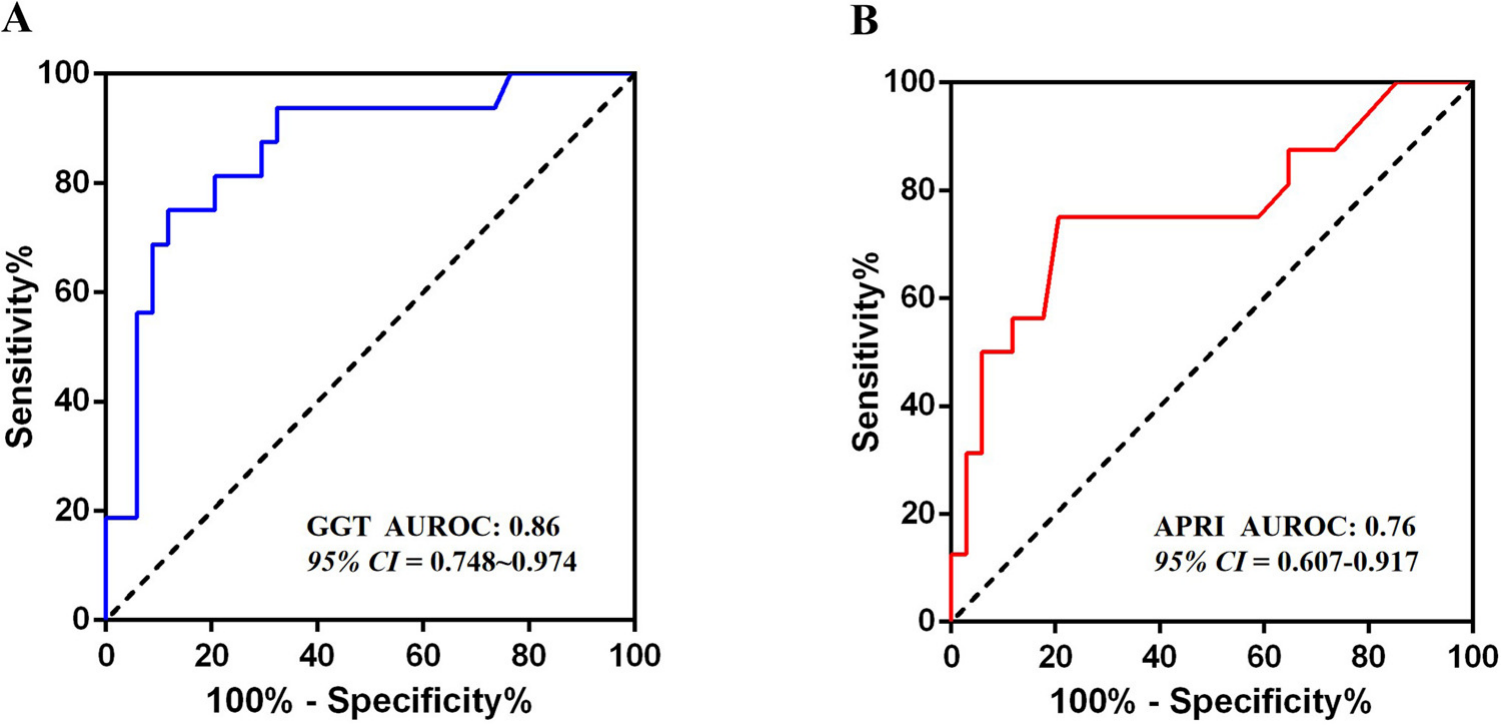
Results of ROC analysis. **(A)** The ROC analysis result of the GGT level; **(B)** The ROC analysis result of the APRI.

Multivariate logistic regression analysis identified Type IV CDCs (OR = 11.55, 95% CI: 1.11–120.63) and elevated GGT levels (OR = 1.003, 95% CI 1.00–1.01) as independent risk factors for significant liver fibrosis in prenatally diagnosed CDCs patients (all *p* < 0.05) (Table 2). GGT exhibited strong diagnostic performance, with an AUROC of 0.86 (95% CI: 0.75–0.97, *p* < 0.001). The optimal cut-off value of 327 U/L provided 75% sensitivity and 88% specificity (Figure 2).

**Table 2:** Multivariate logistic regression analysis of significant fibrosis of prenatally diagnosed CDCs.

Factor	*β*	SE	Wald	*P*	OR	95% CI
Symptomatic	2.548	1.635	2.427	0.119	12.776	0.518∼315.061
Todani classification	2.447	1.197	4.178	0.041*	11.549	1.106∼120.629
AST	−0.001	0.006	0.01	0.921	0.999	0.988∼1.011
GGT	0.002	0.001	4.286	0.038*	1.003	1.000∼1.010
TBIL	0.011	0.006	3.186	0.074	1.012	0.999∼1.024
DBIL	−0.007	0.02	0.115	0.735	0.993	0.956∼1.032
WBC	0.073	0.096	0.584	0.445	1.076	0.892∼1.298
Cyst width	−0.513	0.431	1.417	0.234	0.598	0.257∼1.394

AST, aspartate aminotransferase; GGT, gamma-glutamyl transferase; TBIL, total bilirubin; DBIL, direct bilirubin; WBC, white blood cell count.

* *P* value < 0.05.

### Validation of APRI and FIB-4 in predicting significant liver fibrosis

APRI demonstrated moderate diagnostic utility, with an AUROC of 0.76 (95% CI: 0.61–0.92, *p* < 0.01) (Figure 2). At an optimal cut-off of 0.23, APRI achieved 75% sensitivity, 80% specificity, and 66.7% overall accuracy. In contrast, the FIB-4 showed no significant discriminatory power (AUROC = 0.63, 95% CI 0.45–0.81, *p* = 0.140), with performance statistically comparable to the reference diagonal line.

## Discussion

Hepatic histopathological changes are common in pediatric CDC patients. While gross liver morphology often appears normal, subclinical hepatic injury persists, characterized by biliary ductal hyperplasia, cholestasis, inflammatory infiltration, and periportal fibrosis, with severe cases progressing rapidly to cirrhosis (9). Age-stratified analyses demonstrate increasing fibrosis severity with advancing age, particularly in neonates and infants (9–11). Our cohort showed no significant difference in surgical age between moderate and mild fibrosis subgroups, potentially reflecting population-specific characteristics. Prospective data from Diao et al. suggested accelerated fibrogenesis in early-gestation diagnoses compared to late-gestation cases, implying intrauterine progression of hepatic pathology (2). However, our data showed a non-significant trend toward elevated fibrosis burden in late-gestation diagnoses, leaving the relationship between gestational age at prenatal diagnosis and severe liver fibrosis risk inconclusive. Pathological mechanisms underlying hepatic fibrogenesis in CDCs involve multifactorial processes, including distal biliary obstruction, pancreatobiliary reflux, cholangitis, and biliary hypoplasia (10, 12). In our study, patients with significant liver fibrosis exhibited a higher prevalence of clinical symptoms, particularly jaundice and pale stools. These findings underscore the need for heightened clinical vigilance in prenatally diagnosed CDC neonates presenting with persistent obstructive jaundice, as this may indicate progressive fibrotic changes requiring timely intervention.

Contemporary liver fibrosis staging systems (Scheuer, METAVIR, Ishak, Batts-Ludwig) share a core histological consensus, prioritizing fibrous septa formation as the primary determinant of disease progression (13). Clinically, significant fibrosis (SF) is defined as ≥S2 (Scheuer/METAVIR) or ≥S3 (Ishak), while advanced fibrosis (AF) is classified as ≥S3 (Scheuer/METAVIR) or ≥S4 (Ishak) (14). The Batts-Ludwig system, an optimized iteration of Scheuer's criteria, enhances reproducibility through standardized visual references, making it the gold standard for pediatric hepatic fibrosis assessment (15, 16). In our cohort, 80% of participants exhibited histological fibrosis. The SF rate (32%) and AF rate (10%) were significantly lower than previously reported CDC series (*p* < 0.05), suggesting a relatively mild fibrosis burden in our prenatally diagnosed CDC patients.

This study identifies Type IV CDCs and elevated GGT as independent predictors of significant liver fibrosis in prenatally diagnosed infants, consistent with prior evidence (10, 17). Type IV CDC demonstrates accelerated fibrogenesis due to its unique pathoanatomy, combined intrahepatic/extrahepatic biliary dilatation. The pathophysiology primarily involves mechanical obstruction from protein plugs, biliary sludge, and inflammatory strictures rather than pancreaticobiliary maljunction (17). We speculate that type IV CDCs encompass both intrahepatic and distal bile duct obstructions, driven by protein plugs, bile sludge, inflammatory edema, and congenital strictures. These combined factors accelerate the progression and severity of liver fibrosis in type IV CDCs. Elevated GGT is a biochemical marker of cholestatic injury severity, reflecting obstruction-induced biliary hypertension. Serum GGT level was an independent predictive factor of CDCs perforation (18, 19). Our data support the hypothesis that bile duct obstruction is a key driver of rapid fibrosis progression in infants with prenatal CDC diagnosis.

Developing an accurate, non-invasive diagnostic model for CDC-related liver fibrosis holds significant value for the early diagnosis and treatment of fibrosis in CDC patients. While Tang et al. demonstrated exceptional diagnostic performance of the APRI in pediatric CDC cases (AUROC 0.97, cutoff 0.57, specificity 100%, sensitivity 86.7%) for moderate-to-severe fibrosis (Metavir F3-4), their findings may be constrained by limited sample size, potentially introducing sampling bias (7). Chen et al. proposed a sequential application of Aspartate Aminotransferase-to-Platelet Ratio (APAR) and APRI to optimize diagnostic accuracy, achieving an AUROC of 0.791 with 81.42% sensitivity and 67.65% specificity in progressive fibrosis detection (16). Our cohort analysis reveals that APRI maintains robust predictive value for significant fibrosis in prenatally diagnosed CDC infants. Notably, while liver biochemical markers alone may not comprehensively assess fibrosis extent, GGT levels exceeding 327 U/L exhibit promising discriminative capacity, suggesting its potential integration into multimodal diagnostic algorithms.

The optimal surgical timing for prenatally diagnosed CDCs remains contentious. Current guidelines advocate neonatal intervention for symptomatic cases with jaundice, acholic stools, infection, hepatic dysfunction, or cyst progression (20, 21). However, we believe that in most cases of prenatally diagnosed CDC, the degree of liver fibrosis at birth is not severe. Infants without clinical symptoms can be closely monitored after birth, and surgery may not be necessary during the neonatal period. Conversely, Type IV CDC cases exhibiting biochemical derangements (GGT > 327 U/L) during surveillance warrant expedited intervention due to rapid fibrogenesis risk. It was previously believed that once liver fibrosis develops, it is irreversible; however, increasing evidence shows that fibrosis formation is a dynamic process. Chronic injury leads to both fibrosis deposition and degradation (22). Serial liver biopsies in CDC patients undergoing staged surgical management demonstrated histologic fibrosis regression in 73% (8/11) of cases post-definitive cyst excision (23). Even cirrhotic remodeling shows potential reversibility following timely etiological correction (22). Therefore, once the underlying cause of liver damage is addressed, the majority of CDC patients experience reversible liver tissue pathology, which does not affect their long-term prognosis.

This study has several limitations. Firstly, the small sample size might restrict the robustness of the results, thereby requiring validation with a larger cohort. Secondly, the study was conducted at a tertiary maternal and child health center where the incidence of significant liver fibrosis is relatively high, potentially leading to selection bias. Therefore, multicenter and community-based studies are necessary for further verification. Thirdly, although the current liver fibrosis staging system is widely employed, the definition of stages 2 and 3 remains somewhat subjective. Future research should incorporate artificial intelligence or additional immunohistochemical markers to offer more objective and precise evaluations.

## Conclusion

In conclusion, our cohort observed liver fibrosis predominantly in the early stages (Batts-Ludwig S0∼S1) in 80% of prenatally diagnosed CDC cases. Mechanical bile duct obstruction emerged as the primary pathophysiological driver of accelerated fibrogenesis. The APRI and GGT levels demonstrated strong predictive value for significant fibrosis (AUROC 0.76∼0.86), with GGT > 327 U/L showing 88% specificity. Notably, Type IV CDC cases exhibiting GGT levels exceeding 327 U/L showed an increased risk of significant liver fibrosis, necessitating early surgical intervention. After addressing the underlying cause of liver damage, liver fibrosis in the majority of CDC patients is reversible, and the long-term prognosis is favorable.

## Data Availability

The raw data supporting the conclusions of this article will be made available by the authors, without undue reservation.
